# Role of testosterone in memory impairment of Alzheimer disease induced by Streptozotocin in male rats

**DOI:** 10.1186/2008-2231-20-98

**Published:** 2012-12-23

**Authors:** Pourrabi Seyedreza, Mohajjel Nayebi Alireza, Hossini Seyedebrahim

**Affiliations:** 1 Department of Biology, Science and Research Branch, Islamic Azad University, Fars, Iran; 2 Department of pharmacology and toxicology, Tabriz University of Medical Sciences, Tabriz, 51664, Iran

**Keywords:** Testosterone, Memory, Alzheimer disease, Streptozotocin, Rat

## Abstract

**Background and purpose of the study:**

Recent studies demonstrate that androgens, beyond regulating sexual behavior, exert several neuroprotective functions in the brain. The present study was designed to explore effect of testosterone in memory impairment induced by intra- cerebroventricular (icv) injection of streptozotocin (STZ) as a model of sporadic AD.

**Methods:**

Study was carried out on male Wistar rats. Animals were randomly divided into 11 equal groups. Experimental model of AD was induced by bilateral icv injection of STZ at the dose of 750 μg/Rat/10 μl ACSF at days 1 and 3. STZ-induced memory impairment was assessed two weeks after the last dose of STZ by using a passive avoidance task (1 mA). The interval between the placement of animals in the illuminated chamber and the entry into the dark chamber was measured as a step-through latency (STL). Castration was performed by surgical removing of testis and behavioral study of memory impairment was done after 4 weeks.

**Results:**

Results of this study showed that icv injection of STZ could induce marked (p < 0.05) memory impairment at the dose of 750 μg/Rat/dissolve10 μl CSF/bilateral/days 1 and 3. Therefore, we used this dose of STZ for induction of experimental model of AD. Memory was worsened in castrated rats (P < 0.05) when compared with normal and sham-operated animals. Testosterone replacement therapy (1 mg/kg, sc, for 6 days) in 4 week castrated rats restored memory up to the level of control groups. Testosterone had not any significant effect on memory impairments of non-castrated rats.

**Major conclusion:**

According to the obtained results it can be concluded that testosterone improves cognitive and memory impairment of AD. We suggest that testosterone replacement therapy may have beneficial effect in ameliorating memory impairments of senile patients suffering from AD. Further clinical studies should be carried out to prove possible useful effect of testosterone as an adjuvant therapy in AD.

## Introduction

Alzheimer’s disease is the most common form of dementia in the elderly. The features of Alzheimer’s disease are an amnesic type of memory impairment [1], deterioration of language [2] and visuospatial deficits [3] Motor and sensory abnormalities, gait disturbances, and seizures are uncommon until the late phases of the disease [4]. There is increasing consensus that the production and accumulation of beta-amyloid (Aβ) peptide is central to the pathogenesis of Alzheimer’s disease [5]. Cerebral plaques laden with Aβ and dystrophic neuritis in neocortical terminal fields as well as prominent neurofibrillary tangles in medial temporal-lobe structures are important pathological features of Loss of neurons and white matter, congophilic (amyloid) angiopathy, inflammation, and oxidative damage are also present Alzheimer’s disease [3].

The brain is a well recognized target tissue for androgens [1]. Testosterone (T) acts on neurons and glial cells within multiple sex-hormone sensitive brain areas through both direct effect on androgen receptor (AR) and indirect pathway following aromatization [6]. Regulation of AR protein and AR mRNA by androgens has been observed in mammals in multiple androgen-responsive tissues, such as brain [7]. One of the less known testosterone actions is neuroprotection [8]. In the brain, ARs are expressed by both neurons and glial cells and are mainly found in the thalamus, hypothalamus, hippocampus, amygdale and cerebral cortex where they often co-localize with estrogen receptors (ERs) [9]. During adolescence, T is involved in the activation of preformed brain structures leading to sexually dimorphic physical behavioral and cognitive effects. In adulthood, T exerts neuromodulatory functions that contribute to maintain brain structural and functional homeostasis [1].

T deficiency may be important in patients with Parkinson disease (PD) [10] and Alzheimer disease [11]. It is not well known,however, whether replacement of testosterone in men with borderline hypogonadism and neurodegenerative diseases is of substantial benefit. Androgens significantly modulate specific aspects of cognition, and that androgen depletion, either through normal aging or pharmacological action can result in specific cognitive impairments, increased incidence of neurodegenerative diseases and worse prognosis after brain injury [12]. Brain levels of T were significantly depleted in men with severe AD, indicating that T loss likely precedes development of AD [13]. Clinical studies investigating the effects of testosterone replacement therapy on the cognitive behaviors of healthy older men have produced mixed results, with some studies showing improved spatial ability in men receiving testosterone [14,15], while other studies have shown no effect of testosterone on cognition [14,16]. The results of animal studies are also inconsistent. In various versions of the radial arm maze and T-maze, castration has been shown to impair spatial memory [17,18], whereas other study showed that testosterone replacement in castrated male rats did not restore spatial memory performance on the radial arm maze [19]. These contradictory results highlight the need for the use of rodent models of AD to test the effect of testosterone on memory performance upon other behavioral tests. Therefore, this study designed to investigate role of testosterone in memory impairment induced by streptozotocin (STZ) as a model of sporadic AD by using of passive avoidance test.

## Material and methods

### Chemicals

All chemicals were obtained from sigma except for testosterone enanthate which was purchased from Daroo pakhsh (Tehran, Iran). Solutions were prepared freshly on the days of experimentation.

Testosterone and STZ were dissolved in sterile castor oil and artificial CSF (ACSF: 120 mM NaCl; 3 mM KCl; 1.15 mM CaCl2; 0.8 mM MgCl2; 27 mM NaHCO3; and 0.33 mM NaH2PO4 adjusted to pH 7.2) respectively.

### Animals

The study was performed on male Wistar rats weighting 220–250 g. Animals were housed in standard polypropylene cages, four per cage, under 12:12-h L/D program and at an ambient temperature of 25±2°C, with free access to food and water. Experiments were carried out under the ethical guidelines of the Tabriz University of Medical Sciences for the care and use of laboratory animals.

### Castration

Animals were anesthetized by intraperitoneal (ip) injection of ketamine (60 mg/kg) and xylazine (6 mg kg). The ventral scrotum was shaved and scrubbed with betadine (Behvazan Co, Rasht, Iran). Then 1.5 cm transverse incision was made at midline scrotum; the testes were exteriorized through the incision; the tubules were tied with 0.4 silk suture; and finally testes and testicular fat were removed. The sham surgery consisted of exposing the gonads without removing them. Behavioral studies were done 4 weeks after castration.

### Intracerebroventricular (icv) injection of STZ

Animals were anesthetized with a combination of ketamin (60 mg/kg, ip) and xylazine (6 mg/kg, ip). After they were deeply anesthetized, rats were mounted in a stereotaxic frame in the flat skull position. The scalp was shaved and swabbed with iodine and a small central incision was made to expose the skull. Then two bilateral burr hole were drilled through the skull with the coordinates according to the stereotaxic atlas [20]: anteroposterior from bregma (AP) = −0.8 mm, mediolateral from the midline (ML) = ±1.6 mm and dorsoventral from the skull (DV) = 3.4 mm. STZ (750 μg/10 μl ACSF/Rat, at days 1 and 3) was infused bilaterally into the cerebral ventricles by using a Hamilton syringe and an infusion pump at a flow rate of 0.2 μl/min [19]. Behavioral investigations were carried out 2 weeks after the first injection of STZ.

### Passive avoidance test

The apparatus (Azma Co., Tabriz) consisted of an illuminated chamber connected to dark chamber by a guillotine door. Electric shocks were delivered to the grid floor by an stimulator. On the first and second days of testing, each rat was placed on the apparatus and left for 5 min to habituate to the apparatus. On the third day, an acquisition trial was performed. Rats were individually placed in the illuminated chamber. After a habituation period (2 min), the guillotine door was opened and after the rat entering the dark chamber, the door was closed and an inescapable scrambled electric shock (1 mA, 50HZ, 3 s once) was delivered. In this trial, the initial latency (IL) of entrance into the dark chamber was recorded and rats with IL greater than 60 s were excluded from the study. Twenty-four hours later, each rat was placed in the illuminated chamber for retention trial. The interval between the placement in the illuminated chamber and the entry into the dark chamber was measured as step-through latency (STL_1_, Cut of time 900 s) for short memory. This test was conducted after 3 weeks post-surgery and each rat was tested only once for measured STL_2_ for long memory.

### Statistical analyses

Descriptive staticises and comparisons of differences between means of data sets were calculated by using Instate software. All results were expressed as the mean ± SEM and were analyzed one-way ANOVA. Statistical significance was accepted at the level of P < 0.05. In the case of significant variation (p < 0.05), the values were compared using *post-hoc* Tukey’s test.

## Results

### The effect of STZ on memory impairments

**Figure 1:**
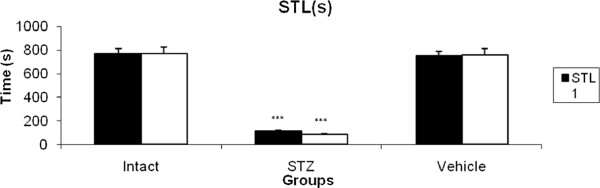
**STl1 (short memory) and STL2 (long memory) in intact, STZ-treated and STZ vehicle-treated rats.** Each bar represents the mean ± SEM of STL (s); n = 8 rats for each group; ***p < 0.001 when compared with intact and STZ vehicle-treated groups.

The Step through latency (STL) was assessed in intact, STZ (750 μg/10 μl ACSF/Rat, at days 1 and 3) and STZ vehicle-treated rats. As it has been shown in Figure 1, the STL1 and STL2 were decreased significantly (P < 0.001) when compared with intact and STZ vehicle-treated animals.

### The effect of castration on STZ induced-memory impairments

**Figure 2:**
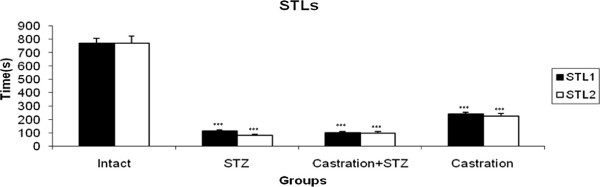
**STl1 (short memory) and STL2 (long memory) in intact, STZ-treated, STZ + Castration and Castration treated rats.** Each bar represents the mean ± SEM of STL (s); n = 8 rats for each group; ***p < 0.001 when compared with intact group.

The STL was assessed in intact, STZ (750 μg/10 μl ACSF/Rat, at days 1 and 3)-treated, Castrated and STZ-treated and castrated animals. As it has been shown in Figure 2, the STL1 and STL2 of STZ, Castration + STZ and castrated groups were decreased significantly (P < 0.001) when compared with intact and STZ vehicle-treated animals.

### The effect of testosterone replacement on STZ induced-memory impairments

Seven groups of rats were shedoulded as: 1) normal rats; 2) testosterone (1 mg/kg, sc, for 6 days)-treated rats; 3) STZ –treated rats; 4) STZ and testosterone (1 mg/kg, sc, for 6 days)-treated rats; 5) STZ-treated and castrated rats; 6) testosterone replacement in STZ-treated and castrated animals; 7) STZ-treated and castrated animals which were treated with vehicle of testosterone.

**Figure 3:**
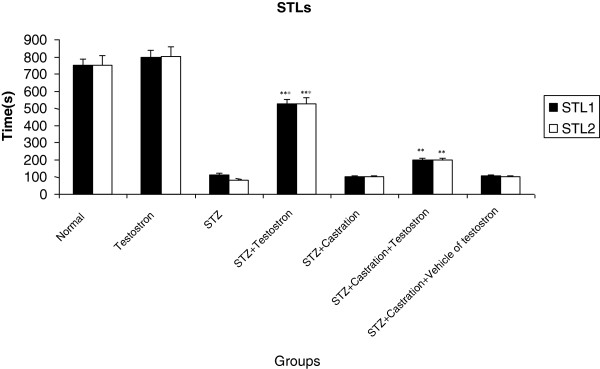
**STl1 (short memory) and STL2 (long memory) in normal, Testosterone, STZ -treated, STZ + Testosterone, STZ + Castration, STZ + Castration + Testosterone and STZ + castration + Vehicle- treated rats.** Each bar represents the mean ± SEM of STL (s); n = 8 rats for each group; ***p < 0.001 when compared with STZ -treated group; **p < 0.01 when compared with STZ + Castration-treated group.

The STL1 and STL2 of testosterone-treated rats were same as the normal animals. In STZ-treated rats which were treated with testosterone, the STL1 and STL2 were significantly (P < 0.001) higher than STZ-treated animals. Furthermore, the amount of STL1 and STL2 were increased significantly (P < 0.01) by testosterone replacement therapy of STZ-treated and castrated rats when compared with also STL was assessed of Testosterone in STZ + Castration with testosterone replacement group was STZ + castration group (Figure 3).

## Discussion

The present study demonstrated that STZ (750 μg/10 μl ACSF/Rat, at days 1 and 3) decreased short (STL1) and long (STL2) memory significantly relative to intact and STZ vehicle-treated. In other words it could cause memory impairment as a symptom of AD. This is in accordance with other reports showed that ICV injection of STZ could induce an experimental model of sporadic AD [21]. This dose of STZ is safe and has been shown do not cause any change in the peripheral blood glucose level [22]. Thus, this protocol was used throughout this study to create animal model of sporadic AD. According to the previous studies, it is known that the effect of testosterone on STZ-induced memory impairment in passive avoidance task has not been studied clearly. Therefore, we used 4-week castrated rats to investigate effect of testosterone depletion on STZ-induced memory impairment. Our results showed that in 4-week castrated rats which were treated with STZ there was a marked memory impairment when assessed by passive avoidance test. This confirms the results of previous study showing that testosterone can have positive activation effects on spatial learning and memory [23]. It has been shown that plasma concentration of testosterone decreases significantly to the undetectable level in 2-week castrated rats [24]. Thus, we can assume that castration-induced memory impairments are related to decrease of serum testosterone levels. Studies indicate that aromatization of androgens to estrogens is a requisite step in some of central action of sex steroids in males of several mammalian species [25]. Estradiol implants were shown to improve spatial memory in castrated rats [26]. Similarly, intra-hipocampal injections of estradiol enhanced the performance of male rats in Morris water maze [27]. Thus the role of this metabolic pathway in memory improving effect of testosterone is not negligible.

Castration-induced memory impairment may be caused indirectly by dysregulation of the hypothalamic-pituitary-gonadal axis. Testosterone normally has negative feedback effects on the brain that keeps levels of gonadotropins relatively low; while castration results in a chronic up regulation of luteinizing hormone (LH) and follicle stimulating hormone (FSH) [28]. Some recent studies have shown that elevated LH impairs spatial memory in female rats [29], and therefore elevated LH may have similar effects in castrated male rats.

In this study testosterone had not any significant effects on STL1 and STL2 of normal non-castrated rats. Thus, it may be assumed that increase of testosterone level to more than physiological amount has not improving effect on memory of normal non-castrated animals. Although testosterone improved markedly short and long term memory of STZ-treated rats and castrated rats which were treated with STZ, but it was not able to restore memory up to the level of normal animals. This could be due to inadequate doses of testosterone.

According to the results, we conclude that castration after 4 weeks diminishes memory in STZ-treated rats, while testosterone replacement therapy attenuates castration-induced memory impairment. We suggest that testosterone may be used as an adjuvant therapy along with other routinely used anti-alzheimer drugs to prevent progressive trends of AD. However, further clinical investigations are needed to prove this hypothesis.

## Competing interest

The authors declare that they have no competing interest.

## Authors’ contributions

PS involved in doing behavioral experiments and drafting. MNA the supervisor of the study participated and involved in concept, design, support of study, interpretation of data and final check of the draft. HS has made contribution in study as an advisor. All authors read and approved the final manuscript.
